# Three-dimensional assessment of the favorability of maxillary posterior teeth intrusion in different skeletal classes limited by the vertical relationship with the maxillary sinus floor

**DOI:** 10.1186/s13005-022-00316-3

**Published:** 2022-04-12

**Authors:** Ehab A. Abdulghani, Abeer A. Al-Sosowa, Maged Sultan Alhammadi, Hanan Al-fakeh, Waseem Saleh Al-Gumaei, Abeer A. Almashraqi, Hasan M. Sharhan, BaoCheng Cao

**Affiliations:** 1 Department of Orthodontics and Dentofacial Orthopedics, College of Dentistry, Lanzhou University, Lanzhou, China; 2 Department of Periodontics, Faculty of Dentistry, Thamar University, Dhamar, Republic of Yemen; 3 Department of Periodontics, College of Dentistry, Lanzhou University, Lanzhou, China; 4 Division of Orthodontics and Dentofacial Orthopedics, Department of Preventive Dental Sciences, College of Dentistry, Jazan University, Jazan, Saudi Arabia; 5 Postgraduate Orthodontic Program, Department of Orthodontics, Pedodontics and Preventive Dentistry, Faculty of Dentistry, Sana’a University, Sana’a, Republic of Yemen; 6 Department of Prosthodontics, School of Stomatology, Lanzhou University, Lanzhou, China; 7 School of Dentistry, Faculty of Dentistry, Ibb University, Ibb, Republic of Yemen; 8 Department of Pre-clinical Oral Health Sciences, College of Dental Medicine, QU Health, Qatar University, Doha, Qatar

**Keywords:** Cone beam computed tomography (CBCT), Maxillary sinus floor (MSF), Posterior maxillary teeth (PMT), Intrusion, Skeletal classes

## Abstract

**Background:**

Understanding the anatomical relationship between the maxillary sinus floor (MSF) and the posterior maxillary teeth (PMT) is important when planning the orthodontic intrusion of the posterior teeth. This study aimed to determine the vertical relationship between posterior maxillary teeth and maxillary sinus floor in different skeletal classes in the Chinese adult population.

**Methods:**

This is a retrospective cross-sectional study involved cone beam computed tomography images of 298 adult patients (145 males and 153 females) between 20 and 45 years old. The sample was categorized according to A point, Nasion, B point (ANB) angle into 102 Class I, 102 Class II, and 94 Class III malocclusion. Non-parametric Wilcoxon Mann–Whitney U and Kruskal–Wallis tests were used to compare the studied groups. The Intra-class Correlation Coefficient (ICC) was used to assess the intra- and inter-observer reliability analysis.

**Results:**

Overall, there was a statistically significant difference in the mean distance between both genders (*P* < 0.001). The measured distance increased with age in all posterior tooth roots (*P* < 0.001). The root apex in the sagittal view appeared to be closer to the maxillary sinus than in the coronal view; 2.2 ± 4.3 and 3.1 ± 5.5 mm, respectively. The most frequent root scores were Type 1 and Type 2P. In both sagittal and coronal views, Class I demonstrated a higher Type 2P prevalence, whereas Class III showed a lower prevalence. The second molars’ mesiobuccal root had the largest number of penetration in the three examined skeletal classes.

**Conclusions:**

Maxillary molars of Class I malocclusion with the majority of Type 2P root-sinus relationship have the highest possible risk of root resorption during molar intrusion due to cortical bone encroachment, while Class III malocclusion showed the least possible risk.

**Supplementary information:**

The online version contains supplementary material available at 10.1186/s13005-022-00316-3.

## Background

The maxillary sinus (MS) is the largest bilateral pyramid-shaped air sinus located in the body of the maxilla. It varies in shape, size, and position on different sides with intra- and inter-individual variations [1]. The maxillary sinus (MS) is the crucial anatomic structure related to the root apices of the posterior maxillary teeth (PMT) and the nasal cavity. The maxillary alveolar process forms the sinus floor, which is located 5 mm below the nasal floor when an individual is about 20 years old [2]. Malocclusions are considered a three-dimensional (3D) problem that requires evaluation in the three planes: (1) anteroposterior, (2) vertical, and (3) transverse planes [3, 4]. Skeletal open bite malocclusion is considered the most challenging malocclusion with respect to its treatment as well as stability [5]. One of the most common protocols to correct this malocclusion is by intruding posterior maxillary teeth [6]. Two main limiting factors for such treatment that might result in apical root resorption of the intruded teeth exist: (1) concentrated orthodontic force over a small apical surface area and, (2) proximity of the teeth to the maxillary sinus with its cortical bone lining.

The maxillary sinus is considered to be an anatomical obstacle for orthodontic tooth movement in both the anteroposterior and vertical directions. Previous research has shown that MS volume pneumatization is not a persistent condition but rather a metabolic process that increases by the age of 12 years and reaches its lowest point around the age of 20 with the complete eruption of the maxillary third molars [7]. The maxillary sinus floor may extend between the posterior maxillary root apices, or sometimes the apices may penetrate into the sinus cavity [8]. However, on histological sections, a thin cortical layer surrounds most of the roots that extend into the sinus, and true perforation rates range from 14–28% [9]. When the posterior teeth intrusion is planned, understanding the anatomical correlation among the posterior maxillary teeth and the maxillary sinus floor (MSF) is important because the close proximity of the two may result in root resorption or delay movement of the tooth during orthodontic intrusion mechanics [10, 11]. The risk of root resorption depends on several factors which including but not limited to the genetic predisposition, the level of the applied force, the surrounding bone anatomy, and the exact distance between the root apex and the cortical bone lining the maxillary sinus.

The distance between the posterior maxillary teeth and the maxillary sinus floor (sinus–apex distance [SAD]) has been evaluated in some studies. Jung et al [12] found that in a sample of Koreans, the maxillary second molars’ mesiobuccal roots were considered the nearest to the sinus floor; however, they did not study age and gender correlations. Von Arx et al. [13] measured the distances between the maxillary sinus and the maxillary premolars’ roots in a Swiss sample and reported that the presence or absence of premolars, gender, age, and side had no significant effects on the mean distances between the sinus floor and premolars roots. The relationship between SADs in the Turkish population was assessed by Kilic et al. [14] and OK et al. [15], both researchers concluded that no significant differences in the right and left sides, but differences in male-female relationships could be found. In contrast, Kilic et al. [14] reported no significant differences between female and male patients, whereas OK et al. [15] found several differences between females and males. Moreover, OK et al. [15] found that depending on the age interval, the relationship between the posterior maxillary teeth root and maxillary sinus floor was different.

For investigating the anatomical correlation between the molars’ roots apices and the floor of the sinus, conventional radiographic examinations, such as periapical and panoramic radiographs, are frequently used [16, 17]. Nevertheless, limits to these two-dimensional (2D) images can be found, which might prevent the proper evaluation of the correlation between the sinus floor and periapical region [16, 18]. Cone beam computed tomography (CBCT) is a 3D imaging technique used in the craniofacial area. Additionally, this method also overcomes the limitations of 2D imaging including magnification, superimposition, distortion and provides multi-planed views [19, 20].

To date, no study has investigated/compared the vertical correlation between the MSF and PMT in different skeletal classes in the adult Chinese population. Therefore, this study aimed to establish this correlation in the three skeletal malocclusions of Chinese adults with possible differences related to age, gender, tooth side, and type of view (sagittal and coronal), based on results from the 3D imaging technique (CBCT).

## Methods

### Subjects

This retrospective and cross-sectional study was approved by the Institutional Ethical Committee (NO LZUKQ-2021-021) at Hospital of Stomatology, Lanzhou University, China. All basic data and CBCT images from February 2016 to March 2021 were evaluated, and patients who fulfilled the inclusion and exclusion criteria were included.

The sample size was calculated with an alpha value of 0.05 and a power of 95% based on a study conducted by Ahn and Park [21] in which the mean distance in the distobuccal root of maxillary first molars in males and females was − 1.80 ± 2.35 and − 0.67 ± 2.42 mm, respectively. The resulting sample size was 117 roots. The minimum number of roots evaluated in any of the sub-groups, including age, gender, skeletal classes, or side, was 256 roots.

The inclusion criteria included: (1) adult patients aged 20–45 years, and 2) presence of upper premolars, and first and second molars with complete root formation. Exclusion criteria were: (1) endodontically treated teeth; (2) history of orthodontic treatment and/or orthognathic surgery; (3) patients with prosthetic crown/s on premolars and/or molars; (4) maxillary sinus lesion and/or periapical lesion of root apices ; (5) defects in the maxillofacial area, such as cleft lip and palate; (6) tempo-mandibular joint (TMJ) disorders, and (7) distorted CBCT images.

**Fig. 1:**
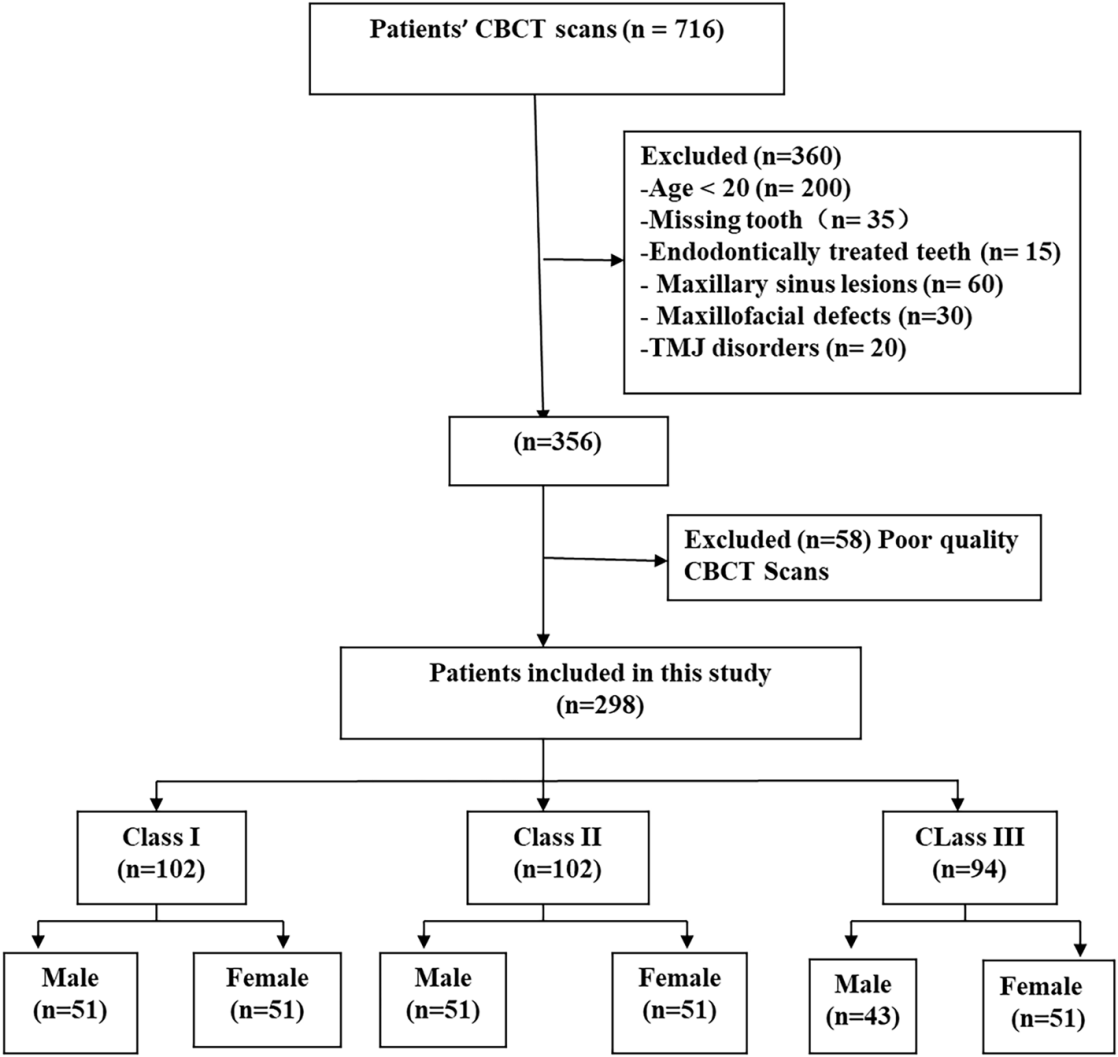
Patient selection flow chart

Data from 716 patients were screened. After applying the selection criteria, 298 (145 males and 153 females) were selected. The sample was categorized according to the ANB angle into 102 Class I, 102 Class II, and 94 Class III malocclusions as presented in (Fig. 1).

### Radiographic acquisition

All CBCT scans were acquired using an i-CAT® imaging device (Imaging Sciences International, Hatfield, PA) with a field of view (FOV) of 16.0 × 13 cm. Scanning parameters were 18.54 mA and 120 kV for a total scan time of 8.9 s with a voxel size of 0.3 mm. The data were stored in Digital Imaging and Communications in Medicine (DICOM) format.

### Three-dimensional measurements

A point, Nasion, B point (ANB) measurements were used to determine the anteroposterior skeletal relationship. The cephalometric radiograph measurements were performed with the InVivo 6.0.3 software program (Anatomage, San Jose, CA). On lateral cephalometric radiographs, several anatomic landmarks were used to classify the skeletal malocclusion: (1) Sella, (2) Nasion, (3) Orbitale, (4) Porion, (5) Point A, (6) Point B, and (7) Pognion. The ANB angle is defined at the intersection of the NA (Nasion-A point) and NB (Nasion-B point) lines. Patients were categorized based on their ANB angles of 0 to 4°, ≥ 4°, and ≤ 0° into skeletal classes Class I, II, and III malocclusions, respectively [21]. Every premolar and molar root was classified into the sagittal and coronal planes based on methods used in previous study [22].

**Fig. 2:**
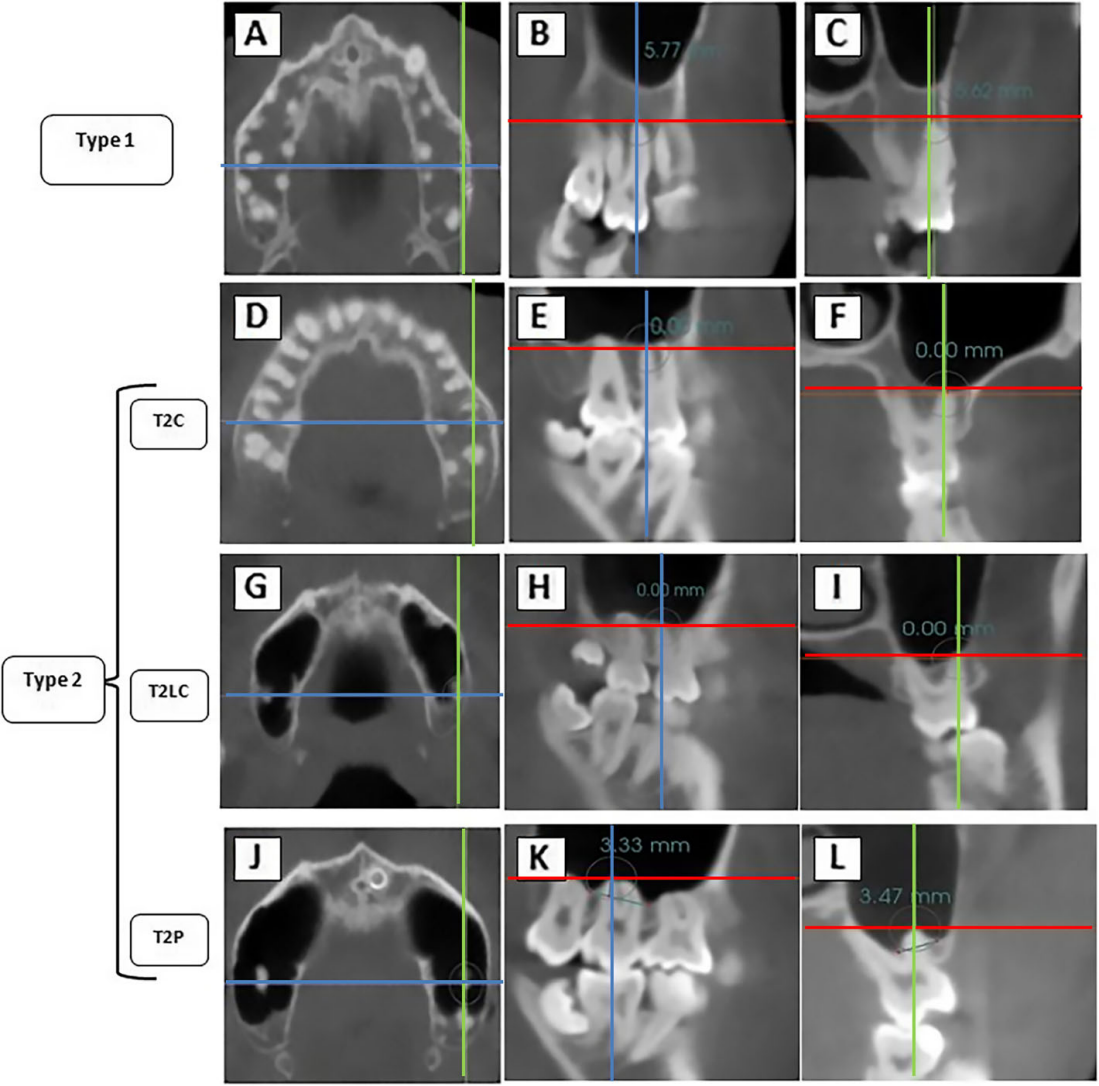
CBCT images demonstrate the three vertical relationships between the maxillary sinus floor (MSF) and the posterior maxillary teeth (PMT). In Axial (**A, D, G,** and **J**), sagittal (**B, E, H,** and **K**) and coronal planes (**C, F, I,** and **L**). Type 1: (**A,B** and **C**); Type 2 C (**D,E** and **F**). Type 2LC (**G, H** and **I**) and Type 2P (**J, K,** and **L**)

**Fig. 3:**
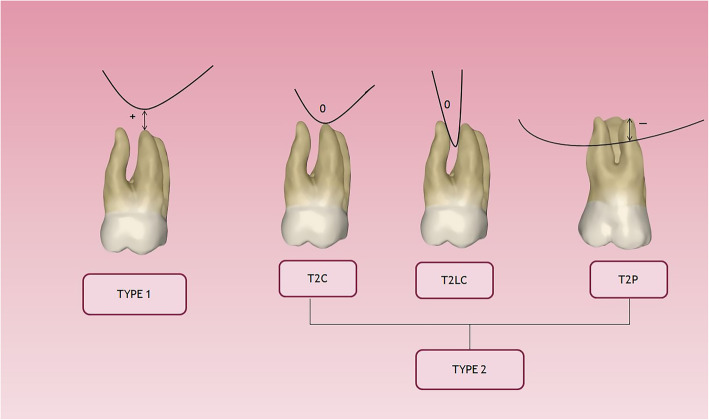
The root apex and the maxillary sinus floor distances

The vertical relationship between the MSF and the PMT was analyzed. When premolars had two roots, the closest root to the MSF was evaluated [22]. For each patient, the maxillary right and left first and second premolars and right and left first and second molars in both the sagittal and coronal views were analyzed. The vertical relationship between the MSF and each root was classified as favorable, indicating no contact (Type 1), or unfavorable, indicating roots in contact (Type 2) for the posterior teeth intrusion (Fig. 2). The relationship in the unfavorable group was sub-divided into three subgroups (T2C, T2LC, and T2P). T2C was considered when direct contact with the MSF occurred, T2LC when the root came into contact with the MSF laterally, and T2P when the root penetrated the MSF [12]. For Type 1, the shortest distance between the roots and the MSF was presented as a positive value; for T2P, it was negative, and T2C, as well as T2LC, were zero (Fig. 3).

### Statistical analysis

All statistical data analyses were performed using the IBM SPSS Version 22.0 (IBM Corp., Armonk, NY). The normality test was performed with the Shapiro–Wilk test. To compare between the studied groups, the non-parametric Wilcoxon Mann–Whitney U and Kruskal–Wallis tests were used. The chi-square test was used to determine distribution differences. To ensure the measurement’s reliability, a random selection of 20% of the selected sample (60 CBCTs) were re-measured by the same observer (Orthodontist; E.A.A) within a 2-week interval in addition to another well trained examiner (Orthodontist; M.S.A) under the supervision and guidance of oral and maxillofacial radiologist with more than 10 years of experience (A.A.A). The intraclass correlation coefficient (ICC) was used to measure intra- and inter-observer agreement. A *P* value < 0.05 was set to indicate statistical significance.

## Results

CBCT radiographs of 298 subjects (Class I = 102 patients [Males = 51; Females = 51], Class II = 102 patients [Males = 51; Females = 51], and Class III = 94 patients [Males = 43; Females = 51]) were evaluated in this study. The mean ages for Class I, II, and III were 28.1 ± 6, 28.4 ± 5.6, and 26.6 ± 5.4 years, respectively. There were 153 (51.3%) females and 145 (48.7%) males with a mean age of 27.3 ± 5.1 and 28.2 ± 6.2 years, respectively. A total of 4768 roots of maxillary posterior teeth (1192 premolars and 3576 molars) were analyzed on the sagittal and coronal views. According to ANB angles,1632 roots were classified as skeletal Class I, 1632 roots as skeletal Class II, and 1504 roots as skeletal Class III malocclusion (Table 1).

**Table 1:** Characteristics of study sample, N (%)

	Total	Class I	Class II	Class III
	***N***=4768	***N***=1632	***N***=1632	***N***=1504
Age group				
20-30	3600(75.5)	1168(71.6)	1184(72.5)	1248(83.0)
>30	1168(24.5)	464(28.4)	448(27.5)	256(17.0)
Gender				
Male	2320(48.7)	816(50.0)	816(50.0)	688(45.7)
Female	2448(51.3)	816(50.0)	816(50.0)	816(54.3)
Type				
Sagital	4768(50.0)	1632(50.0)	1632(50.0)	1504(50.0)
Coronal	4768(50.0)	1632(50.0)	1632(50.0)	1504(50.0)
Tooth side				
Right	2384(50.0)	816(50.0)	816(50.0)	752(50.0)
Left	2384(50.0)	816(50.0)	816(50.0)	752(50.0)
Root type				
U4	596(12.5)	204(12.5)	204(12.5)	188(12.5)
U5	596(12.5)	204(12.5)	204(12.5)	188(12.5)
U6MB	596(12.5)	204(12.5)	204(12.5)	188(12.5)
U6DB	596(12.5)	204(12.5)	204(12.5)	188(12.5)
U6P	596(12.5)	204(12.5)	204(12.5)	188(12.5)
U7MB	596(12.5)	204(12.5)	204(12.5)	188(12.5)
U7DB	596(12.5)	204(12.5)	204(12.5)	188(12.5)
U7P	596(12.5)	204(12.5)	204(12.5)	188(12.5)

The results of the ICC revealed an excellent consistency ranging from 0.984 to 0.990 and 0.948 and 0.976 for intra- and inter-examiner reliability analysis, respectively.

According to the type of view, the mean distance between sagittal and coronal views was significantly different, indicating that the root apex in the sagittal view appeared to be closer to maxillary sinus than in the coronal view (*P *< 0.001; Table 2).

**Table 2:** Differences in the vertical distances related to the multi-planar view and gender

		Mean±SD	Diff. (95% CI)	***P***-value*
View	Sagittal	2.2±4.3	-0.9(-1.1,-0.7)	<0.001
	Coronal	3.1±5.5		
Gender	Male	2.6±5.4	-0.1(-0.3,0.1)	<0.001
	Female	2.7±4.6		

* Mann-Whitney U test was used.

*P-value is considered significant at < 0.05.*

*CI *Confidence Interval.

Generally, on both sagittal and coronal views, the distances in males were significantly shorter than those in females (*P* < 0.001; Table 2).

With regards to age, the distances among the maxillary root apices and the maxillary sinus floor in both sagittal and coronal views increased significantly with age in all posterior tooth roots (*P* < 0.001; Tables 3 and 4).

**Table 3:** The vertical distance between the posterior maxillary roots apices and the maxillary sinus floor related to age, gender, sides, and scores in the sagittal view

		U4	U5	U6			U7			***P***-value^**ª**^
				U6MB	U6DB	U6P	U7MB	U7DB	U7P	
Age	20-30	5.4±4.7	2.4±3.9	1.1±3.3	1.3±3.2	1.4±4.3	0.4±3.2	0.9±3.4	1.8±3.9	<0.001
	>30	8.0±5.1	4.2±4.6	2.8±4.0	2.7±3.7	2.9±4.7	1.3±3.7	2.1±4.2	3.1±4.5	<0.001
	*P*-value*	<0.001	<0.001	<0.001	<0.001	<0.001	0.007	0.002	0.002	
Gender	Male	6.0±5.1	2.7±4.5	1.6±4.0	1.7±3.9	1.5±4.9	0.4±3.8	1.0±4.2	1.8±4.4	<0.001
	Female	6.1±4.7	2.9±3.8	1.5±3.1	1.6±2.9	2.1±3.9	0.8±2.8	1.4±3.0	2.5±3.8	<0.001
	*P*-value*	0.339	0.027	0.117	0.036	0.003	<0.001	<0.001	0.003	
Side	Right	6.0±5.0	2.9±4.2	1.5±3.6	1.6±3.4	1.8±4.5	0.7±3.4	1.2±3.7	2.3±4.2	<0.001
	Left	6.1±4.9	2.8±4.0	1.5±3.5	1.6±3.4	1.8±4.4	0.6±3.3	1.2±3.7	1.9±4.0	<0.001
	*P*-value*	0.621	0.782	0.907	0.824	0.843	0.69	0.735	0.437	
Score	Type 1	6.8±4.7	5.4±3.8	4.2±3.3	4.0±3.4	5.3±3.8	3.7±3.0	4.1±3.4	4.6±3.7	<0.001
	Type 2C	0.01±0.01	0.01±0.01	0.01±0.01	0.01±0.01	0.01±0.01	0.01±0.01	0.01±0.01	0.01±0.01	0.765
	Type 2LC	0.01±0.00	0.02±0.03	0.01±0.00	0.01±0.00	0.01±0.01	0.01±0.01	0.02±0.01	0.01±0.00	0.143
	Type 2P	-1.0±0.5	-1.3±0.8	-1.6±1.2	-1.3±1.0	-2.1±1.4	-2.0±1.3	-1.8±1.2	-2.0±1.4	<0.001
	*P*-value^ª^	<0.001	<0.001	<0.001	<0.001	<0.001	<0.001	<0.001	<0.001	

ª Kruskal-Wallis Test; * Mann-Whitney Test

**Table 4:** The vertical distance between the posterior maxillary roots apices and the maxillary sinus floor related to age, gender, sides, and scores in the coronal view

		U4	U5	U6			U7			*P*-value^ª^
				U6MB	U6DB	U6P	U7MB	U7DB	U7P	
Age	20-30	9.2±8.0	3.2±5.1	1.5±3.6	1.3±3.1	1.6±3.7	0.6±2.9	1.3±3.1	2.4±3.5	<0.001
	>30	13.6±9.3	5.8±6.6	3.5±5.0	2.8±3.9	3.0±4.1	1.6±3.2	2.3±5.2	3.4±4.1	<0.001
	*P*-value*	<0.001	<0.001	<0.001	<0.001	<0.001	0.001	<0.001	<0.001	
Gender	Male	10.6±8.9	3.9±6.2	2.4±4.7	1.8±3.9	1.9±4.3	0.9±3.4	1.4±3.7	2.4±4.0	<0.001
	Female	10.0±8.1	3.8±5.0	1.7±3.3	1.6±2.8	2.0±3.7	0.8±2.5	1.7±3.8	2.8±3.4	<0.001
	*P*-value*	0.741	0.083	0.956	0.166	0.041	0.11	0.001	<0.001	
Side	Right	10.2±8.5	4.0±5.6	2.1±4.2	1.7±3.3	2.0±3.8	0.8±3.0	1.7±3.4	2.8±3.7	<0.001
	Left	10.4±8.5	3.7±5.6	1.9±4.0	1.6±3.4	1.9±3.9	0.8±2.9	1.4±4.1	2.5±3.7	<0.001
	*P*-value*	0.648	0.293	0.545	0.446	0.891	0.532	0.51	0.3	
Score	Type 1	12.0±8.0	7.1±5.5	4.9±4.1	3.9±3.4	4.8±3.6	3.5±2.9	4.0±3.4	4.5±3.5	<0.001
	Type 2C	0.01±0.01	0.01±0.01	0.01±0.01	0.01±0.01	0.01±0.01	0.01±0.01	0.01±0.01	0.01±0.01	0.148
	Type 2LC	0.15±0.35	0.01±0.01	0.01±0.01	0.03±0.11	0.01±0.01	0.01±0.01	0.01±0.01	0.04±0.13	0.89
	Type 2P	-1.2±0.5	-1.2±0.8	-1.5±1.2	-1.4±1.0	-1.5±1.0	-1.6±1.3	-1.5±3.2	-1.1±1.0	<0.001
	*P*-value^ª^	<0.001	<0.001	<0.001	<0.001	<0.001	<0.001	<0.001	<0.001	

ª Kruskal-Wallis Test; * Mann-Whitney Test

Significant differences in Type 1 and Type 2P between the roots in both views could be observed (*P* < 0.001; Tables 3 and 4). According to the skeletal relationship as presented in Table 5, on the sagittal view of Class I, Class II, and Class III first and second premolars, and second molar palatal roots, the Type 1 relationship was more common than Type 2. Second molars’ mesiobuccal and distobuccal roots in all classes showed Type 2 relationships more than Type 1, as well as was positioned in an unfavorable position for orthodontic intrusion. However, only the mesiobuccal and palatal roots of the upper first molar and all upper second molars’ roots showed statistically significant differences (*P* < 0.01).

**Table 5:** Distributions of Type 1 and Type 2 in skeletal Class I, II and III malocclusions

Root type	Score	Sagittal view			***P***-value	Coronal view			***P***-value
		Class I	Class II	Class III		Class I	Class II	Class III	
U4	Type 1	182(34.4)	178(33.6)	169(31.9)	0.635	173(33.7)	173(33.7)	167(32.6)	0.113
	Type 2C	17(34.0)	18(36.0)	15(30.0)		20(32.3)	26(41.9)	16(25.8)	
	Type 2LC	1(11.1)	5(55.6)	3(33.3)		1(16.7)	2(33.3)	3(50.0)	
	Type 2P	4(50.0)	3(37.5)	1(12.5)		10(66.7)	3(20.0)	2(13.3)	
U5	Type 1	120(35.1)	109(31.9)	113(33.0)	0.375	121(35.6)	107(31.5)	112(32.9)	0.006
	Type 2C	30(28.6)	40(38.1)	35(33.3)		38(27.3)	56(40.3)	45(32.4)	
	Type 2LC	11(26.8)	16(39.0)	14(34.1)		2(12.5)	4(25.0)	10(62.5)	
	Type 2P	43(39.8)	39(36.1)	26(24.1)		43(42.6)	37(36.6)	21(20.8)	
U6MB	Type 1	107(37.8)	91(32.2)	85(30.0)	0.007	107(37.2)	86(29.9)	95(33.0)	<0.001
	Type 2C	30(24.0)	39(31.2)	56(44.8)		31(31.0)	38(38.0)	31(31.0)	
	Type 2LC	2(28.6)	4(57.1)	1(14.3)		6(9.4)	32(50.0)	26(40.6)	
	Type 2P	65(35.9)	70(38.7)	46(25.4)		60(41.7)	48(33.3)	36(25.0)	
U6DB	Type 1	102(35.1)	90(30.9)	99(34.0)	0.214	111(37.2)	94(31.5)	93(31.2)	0.004
	Type 2C	42(29.8)	58(41.1)	41(29.1)		30(25.6)	56(47.9)	31(26.5)	
	Type 2LC	0(0.0)	3(60.0)	2(40.0)		12(22.2)	18(33.3)	24(44.4)	
	Type 2P	60(37.7)	53(33.3)	46(28.9)		51(40.2)	36(28.3)	40(31.5)	
U6P	Type 1	93(32.7)	89(31.3)	102(35.9)	0.004	103(34.6)	92(30.9)	103(34.6)	0.01
	Type 2C	24(28.9)	27(32.5)	32(38.6)		19(34.5)	22(40.0)	14(25.5)	
	Type 2LC	1(6.3)	9(56.3)	6(37.5)		11(16.4)	29(43.3)	27(40.3)	
	Type 2P	86(40.4)	79(37.1)	48(22.5)		71(40.3)	61(34.7)	44(25.0)	
U7MB	Type 1	78(32.9)	78(32.9)	81(34.2)	0.001	79(34.2)	80(34.6)	72(31.2)	0.001
	Type 2C	21(24.7)	30(35.3)	34(40.0)		34(25.8)	50(37.9)	48(36.4)	
	Type 2LC	3(11.5)	17(65.4)	6(23.1)		1(4.0)	10(40.0)	14(56.0)	
	Type 2P	102(41.1)	79(31.9)	67(27.0)		90(43.3)	64(30.8)	54(26.0)	
U7DB	Type 1	88(32.0)	95(34.5)	92(33.5)	0.007	100(34.7)	93(32.3)	95(33.0)	0.007
	Type 2C	17(21.8)	28(35.9)	33(42.3)		33(25.6)	56(43.4)	40(31.0)	
	Type 2LC	0(0.0)	0(0.0)	2(100.0)		3(13.0)	9(39.1)	11(47.8)	
	Type 2P	99(41.1)	81(33.6)	61(25.3)		68(43.6)	46(29.5)	42(26.9)	
U7P	Type 1	110(32.2)	115(33.6)	117(34.2)	0.004	129(34.6)	125(33.5)	119(31.9)	0.82
	Type 2C	23(30.7)	28(37.3)	24(32.0)		29(28.4)	39(38.2)	34(33.3)	
	Type 2LC	3(11.1)	10(37.0)	14(51.9)		10(34.5)	11(37.9)	8(27.6)	
	Type 2P	68(44.7)	51(33.6)	33(21.7)		36(39.1)	29(31.5)	27(29.3)	

*Chi-square test*

On the coronal view, most posterior teeth in Class I and Class III were in a favorable position for orthodontic intrusion, which showed Type 1 relationship more than Type 2, except in the second molars’ mesiobuccal root, which was in unfavorable position for orthodontic intrusion while most of the posterior teeth in Class II were in an unfavorable position for intrusion. However, all roots showed statistically significant differences (*P* < 0.01) except for the upper first premolars’ and upper second molars’ palatal roots.

In both sagittal and coronal views, Class I exhibited more Type 2P patterns, whereas Class III exhibited fewer Type 2P patterns compared to other groups. The second molars’ mesiobuccal root had the largest number of penetration in the three examined classes (Table 5).

As shown in Table 6, in both sagittal and coronal views, the upper first premolar was the most root furthest away from maxillary sinus in Class I, Class III, and Class II malocclusion, respectively, while the mesiobuccal root of the second molars considered the most root nearest to the maxillary sinus in the three skeletal classes. Moreover, The most frequent root scores were Type 1 and Type 2P (Table 7).

**Table 6:** Distance differences between the posterior maxillary teeth and the maxillary sinus floor in skeletal Class I, II and III malocclusions

Root type	Sagittal view			***P***-value	Coronal view			***P***-value
	Class I	Class II	Class III		Class I	Class II	Class III	
U4	5.3±4.3	6.8±5.6	6.1±4.7	0.07	9.6±8.3	10.7±8.9	10.6±8.3	0.37
U5	2.3±3.5	3.2±4.7	3.1±4.0	0.176	3.1±4.6	3.9±5.8	4.6±6.3	0.056
U6MB	1.1±3.0	1.7±4.1	1.8±3.5	0.358	1.5±3.5	2.1±4.5	2.4±4.2	0.111
U6DB	1.3±3.0	1.7±3.8	1.8±3.4	0.484	1.3±3.3	1.8±3.4	1.9±3.4	0.488
U6P	1.2±4.0	1.9±4.8	2.4±4.3	0.007	1.3±3.4	2.1±4.1	2.5±3.9	0.009
U7MB	0.4±3.1	0.6±3.4	0.9±3.5	0.135	0.5±3.0	0.9±2.8	1.0±3.1	0.076
U7DB	0.8±3.5	1.3±3.9	1.5±3.6	0.05	1.1±4.4	1.8±3.5	1.7±3.2	0.133
U7P	1.7±3.8	2.2±4.4	2.5±3.9	0.048	2.2±3.5	2.9±3.9	2.8±3.7	0.185

Kruskal-Wallis Test

**Table 7:** Frequencies of root position scores(number and percentage) in the sagittal and coronal views in skeletal Class I, II and III malocclusions

Score	Sagittal view				Coronal view			
	Total	Class I	Class II	Class III	Total	Class I	Class II	Class III
Type 1	2583(54.2)	880(53.9)	845(51.8)	858(57.0)	2629(55.1)	923(56.6)	850(52.1)	856(56.9)
Type 2C	742(15.6)	204(12.5)	268(16.4)	270(18.0)	836(17.5)	234(14.3)	343(21.0)	259(17.2)
Type 2LC	133(2.8)	21(1.3)	64(3.9)	48(3.2)	284(6.0)	46(2.8)	115(7.0)	123(8.2)
Type 2P	1310(27.5)	527(32.3)	455(27.9)	328(21.8)	1019(21.4)	429(26.3)	324(19.9)	266(17.7)

## Discussion

Various complications during orthodontic treatment may occur due to the close anatomic distance between the maxillary sinus floor and posterior maxillary root apices. Hence, understanding the vertical relationship between the MSF and PMT is important when planning the intrusion of the posterior teeth. In this study, the vertical correlation between the MSF and the PMT in the Chinese population in respect to sagittal classification/jaw relationship was evaluated.

In orthodontics, treatment outcome has generally been assessed using 2D analyses, which are performed based on anteroposterior and lateral cephalometric radiographs. It is not easy to estimate 3D root resorption using a two-dimensional (2D) analysis after orthodontic treatment [11, 23]. In multiplanar images, CBCT overcomes the limitations of 2D radiography. Several studies used CBCT to demonstrate the relationship among the maxillary roots and the maxillary sinus floor [12, 21, 23–25]. Nevertheless, most prior reports that assessed these relationships were limited by single-dimensional analysis and small sample size [12, 21, 23–25].

Age results in this study were in agreement with those results from previous studies [26–28], indicating that the distance between the molars apex and maxillary sinus floor increases with age. Tian et al. [29] concluded that mean distances between the sinus floor and the posterior maxillary teeth as well as Type 2P frequency decreased in the Chinese population with increasing ages. Moreover, Gu et al. [28] found that with increasing ages, the distances between the posterior teeth and the sinus floor increased. In contrast, Von Arx et al. [13] reported that age did not affect the distance between the sinus floor and the maxillary premolars, and no significant differences were found between the studied age groups.

Xiaoli HuIn et al. [30] used CBCT scans and found that in males, the posterior maxillary teeth root apices were closer to the MSF than in females; this finding was similar to our findings. On the contrary, other reports in western Chinese [26] and Japanese populations [31] showed that the distance in females was shorter than in males.

The results of the current study showed no significant differences between the distance of maxillary sinus and maxillary posterior root apices on the right and left sides, which was in line with the results of Gu et al. [28], and Zhang et al. [32]. However, our results indicated that the distance at the left side was shorter than that of the right side except for the maxillary first premolars, a finding that was in accordance with the result reported by Pei et al. [26].

The sinus–apex distance on the sagittal view appeared to be shorter than on the coronal view. This finding is consistent with that of Jun Pei et al. [26]. On the other hand, our finding was not in agreement with that of Shuji Oishi et al. [31], this disagreement might be due to differences in the sample size and ethnic group.

Our results showed that the most frequent root scores in terms of skeletal relationships were Type 1. This finding was similar to the previous Chinese publications reported by Jun Pei et al. [26], Gu et al. [28], and Zhang et al. [32]. Furthermore, in all skeletal classes, the intrusion was favorable for the first and second premolars; this finding is in agreement with the studies reported by Ok et al. [15], Eberhardt et al. [33], and Georgescu et al. [34]; in addition, second molar palatal roots were found to be favorable for intrusion, which is in line with reports of Jung et al. [12], and Ok et al. [15] Moreover, the intrusion is mostly unfavorable for the upper second molars' mesiobuccal root.

In the current study, the upper first premolars exhibited the greatest distance, which was similar to the previous findings in Turkish [14], Brazilian [25], Korean [24, 27], and Chinese [28] populations. While the second molars mesiobuccal root of the maxilla exhibited the shortest distance, these findings were agreed with those of previous studies in Brazilian [25], Chinese [26, 29, 32], Korean [27], and American [33] populations. On the other hand, Kilic et al. [14] and Kwak et al. [24] proved that the distobuccal root of the maxillary second molar was the nearest to the sinus floor, which is different from the finding of the current study. However, the study in the Indian population by Kaushik et al. [35] concluded that the palatal roots of the maxillary first molar were the nearest to the maxillary sinus floor. The inconsistencies may be due to differences in the sample size, software used, selected technique, and ethnicity related to molar characteristics.

The three main clinical take home messages of our findings are: (1) during planning for posterior teeth intrusion in adults, evaluation of proximity of root apices and the sinus floor is to be done on both sagittal and coronal views to select the required amount of intrusion with minimum risk, (2) closer eye should be kept when performing posterior maxillary teeth intrusion in adullts, especially in skeletal class I malocclusion male patients to avoid deleterious possible effect, and (3) our findings are applied only for the studied ethnicity and is to be considered with cautions for others.

Study limitations include assessing the differences of distances only in anterioposterior skeletal relationship, the unequal distribution of subjects in the three studied groups (less in class III male patients group). Further studies evaluating these distances in patients with unilateral and/or bilateral skeletal cross bite in addition to increasing the sample size of some groups is recommended.

## Conclusions

Maxillary molars of Class I malocclusion with the majority of Type 2P root-sinus relation showed the highest possible risk of root resorption during molars intrusion due to cortical bone encroachment as the majority of Type 2P root-sinus relation, while Class III malocclusion showed the least possible risk. The highest penetration incidence in the three classes is the maxillary second molars’ mesiobuccal root.

## Supplementary information

**Additional file 1.**

## Data Availability

The datasets used and/or analysed during the current study are available from the corresponding author on reasonable request.

## Abbreviations

MSF: Maxillary sinus floor
PMT: Posterior maxillary teeth
ICC: Intra-class correlation coefficient
MS: Maxillary sinus
2D: Two-dimensional
3D: Three-dimensional
CBCT: Cone-beam computed tomography
SAD: Sinus–apex distance
T2C: Type 2 contact
T2LC: Type 2 lateral contact
T2P: Type 2 penetrate
