# Supplementary material for: Three-dimensional assessment of the favorability of maxillary posterior teeth intrusion in different skeletal classes limited by the vertical relationship with the maxillary sinus floor
Source: Head Face Med. 2022 Apr 12;18:13. doi: 10.1186/s13005-022-00316-3 (PMC9003971; doi:10.1186/s13005-022-00316-3)
Supplement: Supplementary file 1 — Additional file 1. [file 13005_2022_316_MOESM1_ESM.docx]

Dear editor,

We are thankful to you and your reviewers for your time you spent reviewing the manuscript ID: HAFM-D-22-00009 " Three-dimensional assessment of the favorability of maxillary posterior teeth intrusion in different skeletal classes limited by the vertical relationship with the maxillary sinus floor" and the constructive comments that we believe will ultimately improve the manuscript.

We confirm that we have addressed these comments seriously point by point and updated our manuscript accordingly. You will find the changes made highlighted in red color in the revised version. We have included below point-by-point responses to the raised comments as per the journal guidelines.

Thank you.

**Reply to Reviewer #1:**

**General Comment:**

POSITIVE POINTS
- the sample size was calculated
- the inclusion and exclusion criteria were clear
- the figures were clear to explain the distance MSF-PMT

**General Response:**

Thank you so much for the valuable time you spent and the meticulous efforts you did to make this article more clear to the journal readers.

**Comment 1:**

It was not clear how many evaluators have made the measurements (radiologists? Orthodontics?)
**Response 1:**

Thank you for your constructive comment.

It is now mentioned in the revised version in details.

**Comment 2:**

The authors used the classification in Class I, II, and III instead of different vertical growth patterns.

Why were the differences of distances only assessing in anteriorposterior skeletal relationship?

**Response 2:**

Thank you for your kind comment.

Yes the project was of two parts, the first part related to the vertical growth pattern which just published days back by the same research group (<https://link.springer.com/article/10.1007/s00784-022-04458-1> ).

**Comment 3:**

How was the risk ratio of root resorption calculated**?**

**Response 2:**

Thank you for your valuable comment.

The exact risk ratio of root resorption is difficult to be precisely calculated as it is a multifactorial condition but the main factors were mentioned in the revised version.

**Reply to Reviewer #2:**

**General Comment:**

Interesting work.

**General Response:**

Thank you so much for the valuable time you spent and the meticulous efforts you did to make this article more clear to the journal readers.

**Comment 1:**

Please add more clinical relevance of your findings.
**Response 1:**

Thank you for your constructive comment.

More clinical relevance was added.

**Reply to Reviewer #3:**

**General Comment:**

The authors evaluate the relation of posterior teeth roots to the maxillary sinus in respect to the sagittal jaw relationship to assess the favorability of tooth intrusion. The following minor improvements should be implemented:
**General Response:**

Thank you so much for the valuable time you spent and the meticulous efforts you did to make this article more clear to the journal readers.

**Comment 1:**

Page 7, line 43: "... were stored IN Digital ... (DICOM) FORMAT."

**Response 1:**

Thank you for your kind comment.

It was edited accordingly

**Comment 2:**

Page 8, line 1: "... angle is defined AT the intersection ..."

**Response 2:**

Thank you for your valuable comment.

It was edited accordingly

**Comment 3:**

Page 10, line 22 "... Cl I ..." -> "... CL I ..."
**Response 3:**

Thank you for your constructive comment.

The term Class was edited to be full term rather than abbreviated term throughout the revised version.

**Comment 4:**

Page 11, line 15 "... most root away ..." -> ".. root furthest away ..."
**Response 4:**

Thank you for your kind comment.

Done

**Comment 5:**

Page 11, line 17 "... most root near ..." -> ".. root nearest to ..."
**Response 5:**

Thank you for your constructive comment.

Done

**Comment 6:**

Page 11, line 42 "... population in three classes ..." -> "... population in respect to sagittal classification/jaw relationship ..."

**Response 6:**

Thank you for your kind comment.

It was edited accordingly
